# Ion‐ and Temperature‐Programmable Reconfiguration of Subcompartments in Synthetic Cells

**DOI:** 10.1002/cbic.202500928

**Published:** 2026-04-24

**Authors:** Zexi Xu, Petra Schwille

**Affiliations:** ^1^ Department of Cellular and Molecular Biophysics Max-Planck-Institute of Biochemistry Martinsried Germany

**Keywords:** compartment, magnesium, membrane biophysics, synthetic cell, vesicle

## Abstract

Spatial reorganization of subcompartments is a hallmark of living cells, enabling coordinated metabolism and signaling. Achieving dynamic reconfiguration remains a major challenge in synthetic cell design. Although specific ions play fundamental physiological roles, their relevance in bottom‐up synthetic biology has been underexplored. Particularly, magnesium ions (Mg^2+^), essential cofactors and signaling mediators in biological systems, regulate membrane interactions and molecular assemblies. Here, we present a purely Mg^2+^‐mediated mechanism that enables reversible subcompartment assembly within synthetic cells. Mg^2+^ mediates adhesion between negatively charged giant unilamellar vesicles (GUVs), serving as synthetic cell chassis, and neutral large unilamellar vesicles (LUVs), mimicking subcompartments. Mg^2+^ coordination bridges opposing membranes form stable subcompartment layers. Lowering Mg^2+^ concentration, e.g., by chelation with EDTA, disrupts adhesion, whereas reintroduction of Mg^2+^ restores it, enabling dynamic and reversible control over membrane organization. The extent of LUV adhesion depends on membrane charge density, lipid phase state, and temperature. Phase separation allows spatially directed subcompartment to specific domains. Notably, adhesion is lost above the LUV phase transition temperature but re‐established upon cooling, enabling temperature‐programmed reassembly. Together, these findings define a minimal physicochemical framework for dynamic synthetic cell organization through physical stimuli and suggest a primitive lipid–ion mechanism underlying early membrane contact phenomena.

## Graphical abstract

Spatial organization of subcompartments defines living cells, yet achieving dynamic and reversible compartmentalization remains challenging in bottom‐up synthetic cell design. We report a minimal, protein‐free mechanism in which Mg^2+^‐mediated intermembrane adhesion reversibly couples neutral large unilamellar vesicle (LUV) subcompartments to negatively charged giant unilamellar vesicle (GUV) membranes, enabling ion‐ and thermally programmable reorganization of synthetic cell architecture under purely physicochemical control.

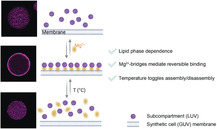

## 1 Introduction

The spatial and temporal organization of subcompartments within cells is highly dynamic. Organelles continuously remodel their contacts to coordinate metabolism, signaling, and trafficking [1, 2, 3, 4]. Spatiotemporal reorganization underlies cellular plasticity, such as synaptic activation [5, 6, 7], intercellular communication [8, 9], lipid exchange, and energy regulation [10, 11]. In these processes, reversible membrane docking and contact formation maintain metabolic homeostasis and ensure spatiotemporal coordination of cellular function. In protocell models, the biomimetic chasses commonly used are giant unilamellar vesicles (GUVs), phospholipid membrane containers that can encapsulate and sustain biochemical reactions. To emulate life‐like behavior of such synthetic vesicular systems, fundamental cellular traits and processes are being reconstructed within, which often require a dynamic spatiotemporal reorganization of subcompartments, being at the core of key physiological functions.

In living cells, most docking processes are orchestrated by highly specialized protein machineries. Complexes such as SNAREs [12], synuclein [13], seipin [14], and small GTPases [15] provide spatial precision and energy to bring membranes into proximity, while tethering proteins and adaptors stabilize the resulting contact sites. However, there is evidence from synthetic and cell‐free systems that membranes can also adhere in the complete absence of proteins when divalent cations such as magnesium (Mg^2+^) or calcium (Ca^2+^) are present [16, 17, 18, 19, 20, 21]. Moreover, Mg^2+^, the most abundant divalent ion in cells, plays a central role in biology, as an essential cofactor for many enzymatic and signaling processes [22, 23, 24, 25], and is therefore an obvious constituent of most synthetic cell systems. What remains largely unexplored to date, however, is how these fundamental electrostatic mechanisms can be harnessed in low‐complexity minimal cells to achieve programmable control over subcompartment organization. In particular, the combined tuning of membrane composition, ionic environment, and lipid phase state to construct multicompartment architectures with reversible and stimulus‐responsive behavior has not yet been systematically demonstrated, while previous efforts have primarily focused on assembling hierarchical structures through direct electrostatic pairing of oppositely charged membranes or via chemical reactions [26, 27, 28, 29, 30, 31, 32, 33].

In addition, lipid structure critically determines how membranes interact with ions. Lipids differ in shape, headgroup charge, and acyl‐chain length and saturation that define the physical state of the membrane and thereby its biological function. Saturated phospholipids and glycosphingolipids preferentially partition into tightly packed, liquid‐ordered (Lo) domain, while unsaturated lipids favor more fluid, liquid‐disordered (Ld) domain [34, 35]. Differences in lipid composition and domain organization strongly influence how ions interact with membranes, thereby shaping electrostatic phenomena such as DNA–lipid association [36, 37, 38, 39]. Biological evidence further supports a link between lipid order and organelle docking efficiency [40, 41, 42, 43]. These examples suggest that membrane phase and local ionic conditions can cooperate with protein machineries to modulate the efficiency and selectivity of membrane docking. However, disentangling these coupled physicochemical contributions within living cells is challenging because of highly interdependent protein functions, cytoskeletal forces, and complex ionic environments.

In this study, we establish a minimal synthetic cell model based on GUVs capable of reversible and spatially defined adhesion of large unilamellar vesicles (LUVs) as subcompartments. By systematically tuning membrane composition and ionic environment, we separated and dissected the physicochemical principles governing subcompartment assembly driven solely by lipid bilayers and physiologically relevant ions. Specifically, we asked the following questions: (1) Does Mg^2+^ mediate reversible adhesion between anionic synthetic cells and neutral subcompartments? (2) Is the adhesion selective and spatially programmable? (3) Can this Mg^2+^‐mediated adhesion be dynamically regulated by external physical cues such as temperature? Here, we use the term “subcompartment” broadly to describe the hierarchical organization of LUVs relative to the GUV chassis, encompassing both internal encapsulation and external surface adhesion, as both represent subordinate compartmental units within the synthetic cell architecture. Our findings define a purely physicochemical framework for membrane contact site formation and demonstrate that complex spatial reorganization may emerge from the synergistic interplay between lipid composition, phase state, and Mg^2+^, providing new design principles for constructing programmable and responsive synthetic cells.

## 2 Results and Discussion

### 2.1 Mg^2+^‐Mediated DPPC LUVs Binding to GUVs

**FIGURE 1:**
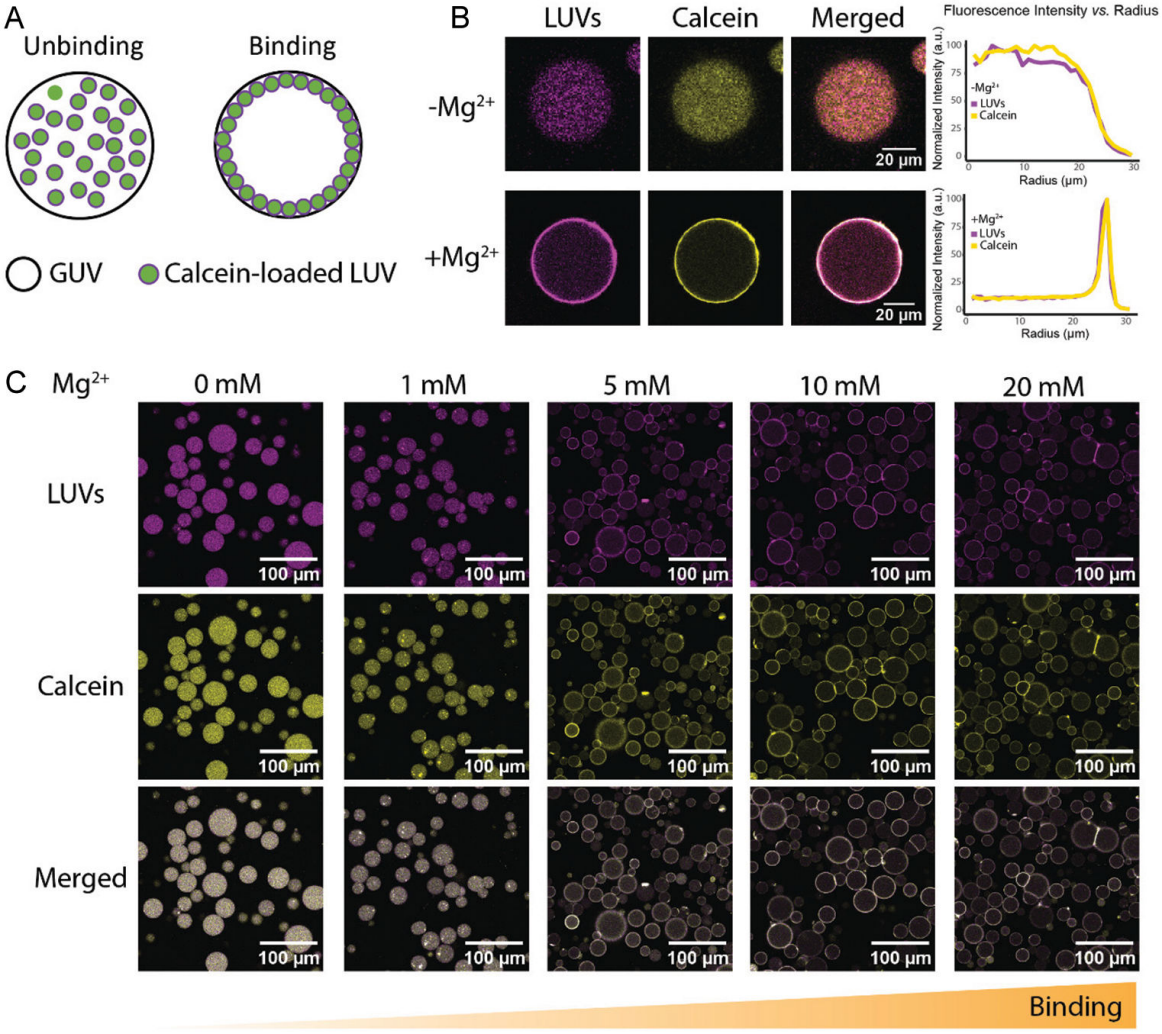
Mg^2+^‐mediated binding of subcompartments to synthetic cell membranes. (A) Schematic illustration of artificial cells showing two states: dispersed subcompartments within the lumen (left) and membrane‐bound subcompartment layers formed by liposome (LUV)–giant unilamellar vesicle (GUV) adhesion (right). (B) Confocal images of calcein‐loaded DPPC LUVs labeled with Atto655‐DPPE in the absence and presence of 5 mM Mg^2+^. Without Mg^2+^, LUVs remain dispersed in the GUV lumen, whereas 5 mM Mg^2+^ induces their adhesion to the GUV membrane, forming a continuous layer without fusion. GUVs were composed of POPC/POPG (70/30 mol%). Normalized fluorescence intensity profiles as a function of distance from the GUV center, showing LUV distribution in the absence (0 mM) and presence (5 mM) of Mg^2+^ (right). Peaks at the membrane indicate LUV adhesion. (C) Increasing Mg^2+^ concentration strengthens LUV–GUV association, with no apparent fusion, as calcein fluorescence remains colocalized with the LUV signal rather than diffusing into the lumen. Magenta: LUVs; yellow: calcein.

To test whether and how Mg^2+^ can mediate protein‐free subcompartments to adhere to synthetic cells, we reconstituted a minimal multicompartment system in which neutral, calcein‐loaded LUVs composed of DPPC lipid interacted with negatively charged GUVs (POPC/POPG, 70/30 mol%). Negatively charged GUV served as the synthetic cell chassis, while neutral, organelle‐like LUVs acted as subcompartments, a term we use to describe LUVs in hierarchical relationship to the GUV chassis, whether encapsulated internally or adhered externally. In the absence of Mg^2+^, DPPC LUVs were homogeneously dispersed within the GUV lumen without forming membrane contacts. Upon addition of 5 mM Mg^2+^, hierarchical architectures formed in which neutral DPPC LUVs adhered to the negatively charged GUV membranes (Figure 1B). Increasing Mg^2+^ concentration from 0 to 20 mM progressively strengthened this association (Figure 1C). At 1 mM Mg^2+^, no stable subcompartment layer was observed, although small fluorescent patches suggested occasional transient contacts between LUVs and the GUV membrane. At 5 mM Mg^2+^, a continuous LUV adhesion layer formed, which remained stable up to 20 mM Mg^2+^ without detectable LUV fusion. Notably, this concentration range corresponds to physiological Mg^2+^ levels in living cells, which can reach approximately 20 mM, though free Mg^2+^ is typically lower (∼0.5–1 mM) [24, 44]. The concentrations used here may reflect the absence of competing Mg^2+^‐binding biomolecules in our minimal system. Mg^2+^ was introduced during GUV formation using the phase‐transfer encapsulation method, such that Mg^2+^ was present in the inner aqueous phase of the GUVs together with the encapsulated LUVs. When the Mg^2+^ concentration reaches at 60 mM Mg^2+^, subcompartment layer is markedly suppressed. As these conditions lie far beyond physiological ionic ranges, it is not discussed further in this work. Calcein was encapsulated inside the LUVs to monitor potential fusion events. If Mg^2+^ induced fusion between LUVs and GUV membranes, the encapsulated calcein would have been released into and diffused throughout the GUV lumen. However, in our experiments, calcein fluorescence remained colocalized with the LUV membrane signal across all Mg^2+^ concentrations we used (Figure 1B,C). This persistent overlap confirms that Mg^2+^ mediates membrane adhesion without fusion, indicating that subcompartments remain intact and physically associated with the GUV surface through nonfusogenic adhesion. Calcein itself does not adhere to POPC/POPG membranes in the presence of 5 mM Mg^2+^ (Figure S1) and similarly shows no binding to DPPC membranes under the same conditions (Figure S2). The hydrodynamic diameter of DPPC LUVs remained constant (∼159 nm) across 0–20 mM Mg^2+^, indicating no aggregation. The polydispersity index (PDI) showed an increase at 1 mM Mg^2+^ but decreased at higher concentrations, suggesting that the observed membrane adhesion at ≥5 mM Mg^2+^ does not result from vesicle aggregation in solution, but rather from stable LUV–GUV surface binding (Figure S3A).

To further substantiate the Mg^2+^‐mediated interaction between LUVs and GUVs, we measured the zeta potential of zwitterionic DPPC and anionic POPC/POPG (70/30 mol%) membranes, which revealed distinct Mg^2+^‐dependent surface charge responses (Figure S3B). At 0–1 mM Mg^2+^, both DPPC and POPC/POPG vesicles exhibited negative surface potentials, preventing electrostatic attraction and consistent with the absence of subcompartment layer formation. Increasing Mg^2+^ to 5 mM caused the DPPC vesicles to shift to a positive potential (+4.6 ± 0.1 mV) in agreement with previous studies [36, 45], while POPC/POPG remained negative (−15.3 ± 0.0 mV), enabling electrostatic attraction between the two membranes and subcompartment assembly. Further elevation to 20 mM Mg^2+^ increased the surface potentials to + 11.4 ± 0.6 mV (DPPC) and −5.0 ± 0.7 mV (POPC/POPG). Mg^2+^ coordination with the negative phosphate groups of zwitterionic DPPC [36, 46], combined with headgroup reorientation [47], results in a net positive surface potential.

At low ionic strengths, phosphate groups are positioned toward the outer headgroup region, exposing negative charge [47, 48, 49]. Upon Mg^2+^ binding and increased ionic strength, the positive choline moieties are driven outward while phosphate groups are buried beneath the bilayer surface. This conformational change, together with Mg^2+^ coordination, shifts the zeta potential from negative (−3.7 ± 1.8 mV at 0 mM Mg^2+^) to positive at 5 mM Mg^2+^, enabling electrostatic attraction to negatively charged membranes. In contrast, Mg^2+^ interacts with negatively charged POPG headgroups primarily through water‐bridged coordination [50]. This interaction partially neutralizes the surface charge of POPC/POPG membranes from −27.4 ± 0.7 mV at 0 mM Mg^2+^ while maintaining sufficient negative charge to attract the positively charged DPPC LUVs. Note that all buffers used in this system contained monovalent ions 150 mM KCl and 25 mM Tris to mimic physiological conditions and maintain a stable pH (∼7.5). However, no subcompartment layer formation was observed in the absence of Mg^2+^, indicating that the observed behavior arises from Mg^2+^ bridging. Together, these processes establish an electrostatic asymmetry that drives adhesion of subcompartment layers. Previous studies have shown that high KCl concentrations (100 mM) can disrupt electrostatic interactions between negatively charged DOPG‐containing subcompartments and positively charged DOTAP‐containing membranes [26]. In our system, this disruption was not observed, indicating that Mg^2+^‐mediated adhesion remains stable under conditions that more closely mimic physiological ionic strengths and can be integrated with a broad range of experimental setups in synthetic cell systems where high KCl concentrations are employed [51, 52, 53, 54].

### 2.2 Time‐Dependent Assembly of Mg^2+^‐Mediated Subcompartment Layers

**FIGURE 2:**
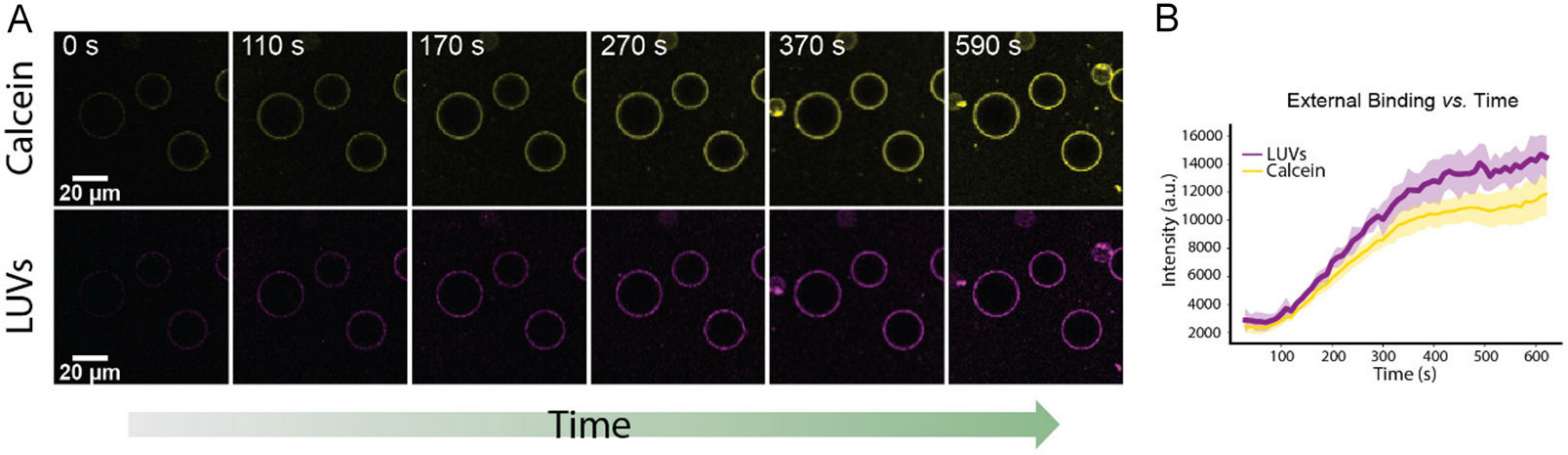
Kinetics of subcompartment layer assembly. (A) Time‐dependent association of DPPC LUVs with the outer surface of GUVs. (B) Quantification of LUV membrane fluorescence intensity at the GUV surface as a function of time.

To examine the dynamics of Mg^2+^‐mediated docking, calcein‐loaded DPPC LUVs were first added to the exterior of POPC/POPG (70/30 mol%) GUVs, after which Mg^2+^ was introduced into the system to a final concentration of 5 mM. The time series began recording less than a minute after LUV addition, and time zero corresponds to the start of imaging. Confocal data (Figure 2A, Video S1) showed that the fluorescence signal from LUVs progressively increased on the GUV surface over time, with the encapsulated calcein fluorescence remaining colocalized with the LUV signal throughout the experiment, confirming docking without fusion. Quantitative analysis of fluorescence intensity (Figure 2B) revealed a gradual increase in surface binding that reached a plateau after approximately 300 s, indicating a time‐dependent adsorption process that stabilizes once the available binding sites are saturated under steady Mg^2+^ bridging conditions.

### 2.3 Mg^2+^‐Mediated Reversible Outer Subcompartment Layer Assembly

**FIGURE 3:**
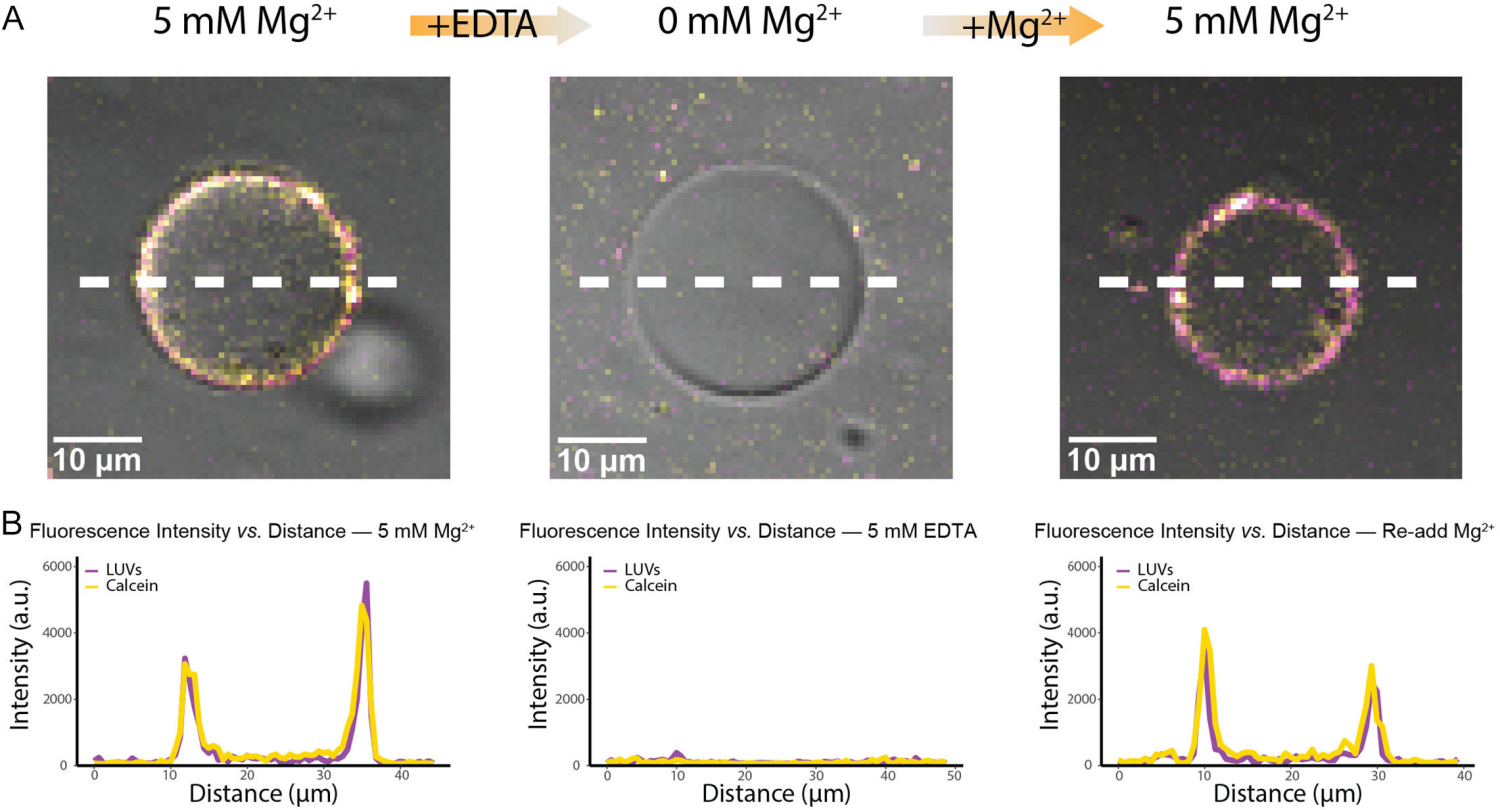
Subcompartment layer assembly and disassembly by changing the Mg^2+^ concentrations. (A) Confocal images showing DPPC LUVs (magenta, Atto655‐DPPE) loaded with calcein (yellow) on the outer surface of POPC/POPG (70/30 mol%) GUVs. Reversible assembly and disassembly of LUV layers controlled by Mg^2+^ and EDTA. DPPC LUVs initially form a membrane‐bound layer on the outer surface of GUVs; subsequent addition of EDTA disrupts Mg^2+^‐mediated interactions, leading to LUV detachment. Reintroduction of Mg^2+^ restores LUV binding. (B) Fluorescence intensity profiles as a function of distance from the GUV center, showing the three states: initial LUV binding with 5 mM Mg^2+^ (left), LUV detachment after EDTA addition (middle), and LUV reassembly after Mg^2+^ readdition (right). Peaks at the membrane position indicate LUV localization; their disappearance and reappearance confirm reversible adhesion control.

The electrostatic interactions between membranes allowed controlled dispersal of the outer subcompartment layers by reducing the Mg^2+^ concentration. To test whether Mg^2+^‐mediated adhesion is reversible, we used EDTA to chelate Mg^2+^ ions and disrupt membrane bridging. We added 5 mM EDTA matching the 5 mM Mg^2+^ initially present. Confocal imaging revealed that this treatment chelated Mg^2+^ ions, disrupting the electrostatic bridges between membranes and causing the subcompartment layers to detach from the outer GUV surface (Figure 3A). Importantly, the process was reversible. Reintroduction of 5 mM Mg^2+^ restored LUV membrane bridging to the GUV and reassembled the outer subcompartment layer. The overall procedure is summarized in Figure S4. Briefly, EDTA was incubated with LUV‐coated GUVs for 5 min in an Eppendorf tube prior to imaging. MgCl_2_ was then added to the EDTA‐treated sample and likewise incubated for 5 min before imaging. As shown in Figure 2B, binding reaches a plateau after approximately 5 min, indicating that this timescale is sufficient for both detachment and reattachment in response to Mg^2+^ concentration. Quantitative analysis showed that the reassembled layer recovered (Figure 3B), demonstrating that EDTA‐mediated detachment does not irreversibly damage the membranes. This reversibility shows that it is possible to dynamically regulate synthetic cell architecture simply by adjusting Mg^2+^ concentrations, mirroring aspects of biological membrane contact dynamics, e.g., when organelles establish and dissolve contacts in response to metabolic state, Ca^2+^/Mg^2+^ fluctuations, or signaling events [4, 13, 43]. While biological systems employ protein tethers and active transport, our minimal system demonstrates that ionic regulation alone can achieve spatial reorganization. This control mechanism has potentially been accessible to early protocells and may be relevant for pathological conditions where protein machineries are compromised.

### 2.4 Mg^2+^‐Mediated Localized Multicompartment Structure

**FIGURE 4:**
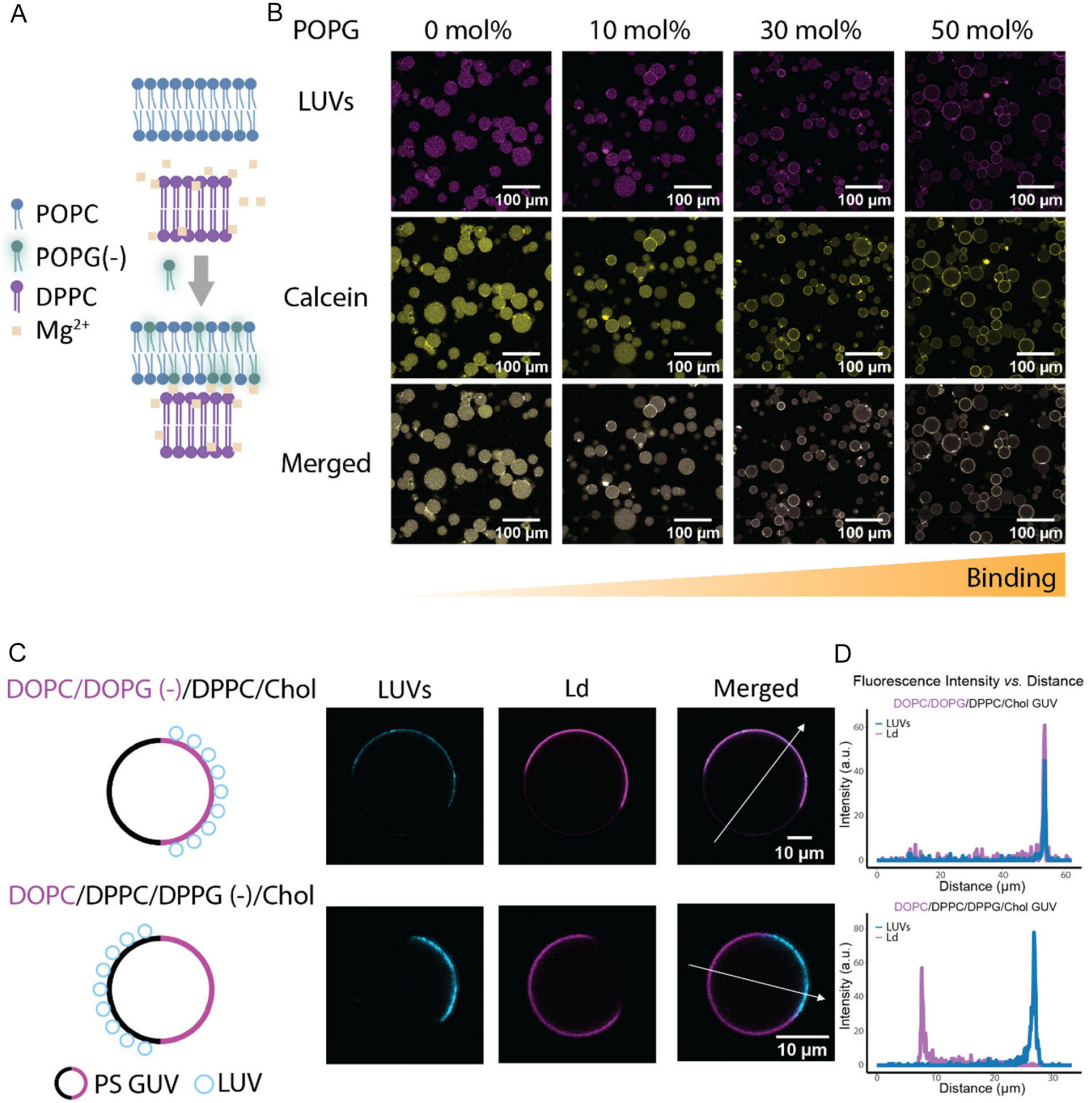
Membrane surface charge governs subcompartment positioning in synthetic cells. (A) Schematic illustration showing how synthetic cell membrane surface charge influences subcompartment association. Created in BioRender. Xu, Z. (2026) https://BioRender.com/atohcuq. (B) Increasing the fraction of negatively charged lipid (POPG) in the GUV membrane enhances the adhesion of subcompartments to the GUV surface (Mg^2+^: 5 mM). Magenta: LUVs; yellow: calcein. (C) Schematic (left) and confocal images (right) of phase‐separated synthetic cells showing LUV association with charged membrane domains. When negative charge is in the Ld phase, LUVs (blue) preferentially bind to Ld regions (magenta); conversely, when charge is in the Lo phase, LUVs associate with Lo domains. (D) Fluorescence intensity profiles indicate colocalization of LUV and GUV domain under each condition.

Having confirmed that Mg^2+^ mediates reversible subcompartment positioning through ion‐bridge formation, we next examined how the surface charge of the synthetic cell membrane modulates LUV adhesion. Figure 4A illustrates the concept that increasing negative surface charge enhances electrostatic attraction to zwitterionic DPPC LUVs in the presence of Mg^2+^. Consistent with this, confocal images in Figure 4B show that the extent of LUV binding increases with the POPG fraction (0–50 mol%) in the GUV membrane at the same Mg^2+^ concentration (5 mM). Zeta potential of POPC/POPG as a function of POPG is shown in Figure S5. The statistics of GUVs displaying DPPC subcompartment layer at different POPG concentration is shown in Figure S6. These results establish membrane charge density as a key parameter for controlling the extent of LUV binding.

To control the spatial distribution of subcompartments, we next introduced phase‐separated GUVs in which charge is confined to specific membrane domains. Phase‐separated GUVs composed of DOPC/DPPC/Cholesterol exhibit coexisting liquid‐disordered (Ld) and liquid‐ordered (Lo) domains at room temperature [34, 55], where DOPC partitions into the fluid Ld phase and DPPC together with cholesterol forms the more rigid Lo phase. The Ld phase was fluorescently labeled with Atto655‐DOPE, while the unlabeled Lo phase remained dark, allowing clear visualization of phase distribution. To localize charge within specific membrane phases, DOPG was incorporated when the negative charge was supposed to be in the Ld domain (DOPC/DOPG/DPPC/Chol/Atto655‐DOPE 29.95/10/40/20/0.05 mol%), and DPPG was used when charge localization in the Lo domain was desired (DOPC/DPPC/DPPG/Chol/Atto655‐DOPE 40/29.95/10/20/0.05 mol%). When charge heterogeneity was introduced in phase‐separated GUVs, LUVs preferentially accumulated at the negatively charged domains (Figure 4C,D), indicating that membrane charge distribution can regulate the spatial organization of subcompartment adhesion. This controlled adhesion may be useful to engineer synthetic cells with programmable spatial organization in which the positioning of subcompartments are required to unfold key cellular functions [6, 7].

### 2.5 Mg^2+^‐Mediated Selective Binding

**FIGURE 5:**
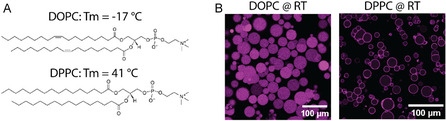
Subcompartment membrane composition determines subcompartment positioning. (A) Chemical structures of DOPC and DPPC used to form distinct subcompartments. (B) Confocal images show that Atto655‐DOPE–labeled DOPC LUVs fail to bind to negatively charged GUVs (POPC/POPG, 70/30 mol%), whereas Atto655‐DPPE‐labeled DPPC LUVs adhere under the same Mg^2+^ concentration (5 mM) at room temperature (RT: 19°C–25°C).

To examine whether Mg^2+^‐mediated adhesion can be used for selective binding, we compared LUVs composed of different neutral zwitterionic lipids: DOPC and DPPC, which share the same phosphocholine headgroup but differ in acyl chain length and saturation. DOPC, containing two unsaturated tails with a transition temperature (*T*_m_) of −17°C, forms a fluid‐phase membrane at room temperature, whereas DPPC, with fully saturated tails and a transition temperature of 41°C, remains in the gel phase at room temperature [56, 57]. The transition temperature defines the point at which a lipid bilayer shifts from an ordered, tightly packed gel state to a disordered, fluid state, thereby determining its rigidity and packing density. Confocal imaging revealed that at 5 mM Mg^2+^, DOPC LUVs did not dock to negatively charged POPC/POPG 70/30 mol% GUVs, whereas DPPC LUVs formed a clear membrane‐attached layer (Figure 5). Dynamic light scattering (DLS) confirmed that both lipids formed vesicles of similar size and homogeneity, with DOPC LUVs measuring 145.9 ± 2.8 nm with a PDI of 0.161 ± 0.011 and DPPC LUVs measuring 155.0 ± 2.8 nm with a PDI of 0.167 ± 0.021 as shown in Figure S7A. These data rule out size effects as the cause of the binding difference.

The selective docking behavior likely arises from differences in membrane phase state and lipid packing. The gel‐phase DPPC bilayer, with its tightly organized structure and reduced lateral lipid diffusion, can maintain stable Mg^2+^‐mediated contacts once formed at the GUV surface. In contrast, the fluid‐phase DOPC bilayer’s high lateral mobility may prevent the formation or maintenance of stable contact sites, as Mg^2+^‐coordinated lipid clusters can rapidly reorganize and dissipate [36, 37]. Consistent with this interpretation, zeta potential measurements showed that DPPC LUVs exhibited a slightly more positive surface potential (+4.1 ± 0.3 mV) compared to DOPC LUVs (+0.2 ± 0.8 mV) in the presence of 5 mM Mg^2+^ (Figure S7B), suggesting enhanced Mg^2+^ coordination on the more ordered DPPC surface [37, 39]. This difference in zeta potential may suggest that membrane phase state and packing dynamics, rather than simply the extent of Mg^2+^ binding, play the dominant role in determining adhesion. Consequently, Mg^2+^ ions selectively mediate docking between gel‐phase subcompartments and negatively charged synthetic cells, while fluid‐phase subcompartments remain nonadhesive under the same ionic conditions. These data provide a basis for temperature‐controlled reversibility described in the following section.

### 2.6 Temperature‐Mediated Reversible Subcompartment Layer Assembly

**FIGURE 6:**
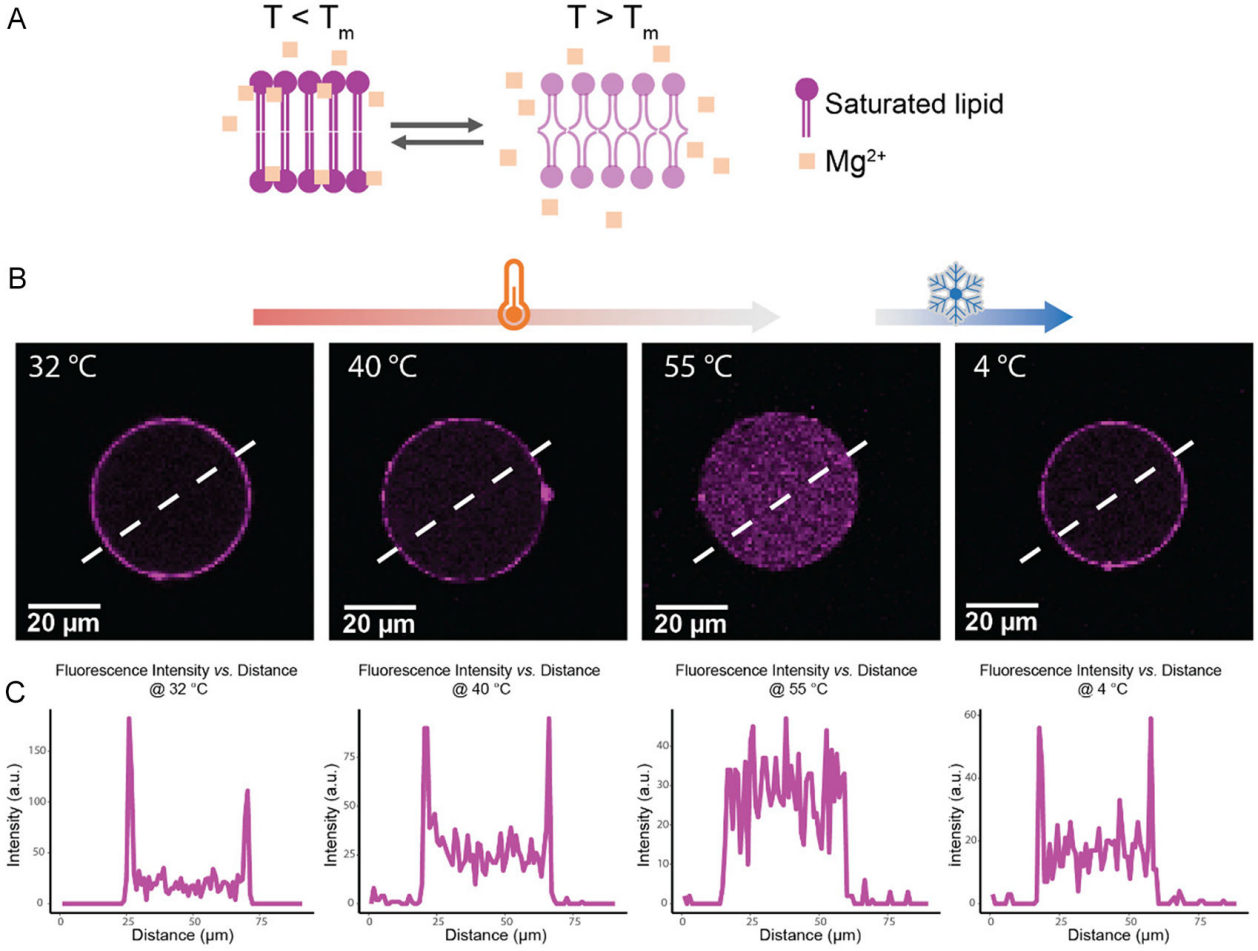
Temperature‐dependent reversible assembly of subcompartment layers. (A) Schematic illustration of Mg^2+^ interactions with DPPC membranes below and above the lipid phase transition temperature (*T*_m_). (B) Temperature‐controlled reversible binding of DPPC LUVs to GUVs. Above the DPPC phase transition temperature (55°C), LUVs dissociate from the GUV membrane; upon cooling to 4°C, LUVs re‐associate. LUVs are shown in magenta. (C) Fluorescence intensity profiles showing subcompartment assembly, disassembly, and reassembly states in response to temperature.

To gain an additional physical control over Mg^2+^‐mediated adhesion, we tested whether temperature could reversibly regulate the attachment of DPPC subcompartments to the GUV membrane, building on our finding that lipid phase can be used for selective binding. The phase state of DPPC is strongly temperature‐dependent, with gel‐to‐fluid transition occurring at around 41°C. Below this transition temperature, the rigid lipid membrane efficiently coordinates Mg^2+^ ions. Above the transition temperature, the fluid membrane has low affinity of Mg^2+^ for the lipid headgroups [37, 58, 59, 60]. As illustrated in Figure 6A, Mg^2+^ ions are absorbed on the ordered DPPC membrane below *T*_m_, but dissociate when the lipid is fluid phase. In addition, lipid headgroup orientation changes with increasing temperature. At the phase transition temperature of the lipids, the negative phosphate moiety protrudes outward while the positive choline retreats inward [48]. This conformational change together with reorganization of ion binding increases negative surface potential [37, 58, 59].

Confocal data demonstrated that DPPC LUVs adhered to the negatively charged POPC/POPG (70/30 mol%) GUVs below phase transition temperature, but completely detached at 55°C. When the temperature was subsequently lowered to 4°C, the subcompartments reassembled into a clear surface layer, indicating that the process is reversible (Figure 6B, Video S2). The corresponding fluorescence intensity profiles across the GUV membrane (Figure 6C) confirmed these observations, showing pronounced peaks at the membrane interface at 32°C and 40°C, a flattened distribution at 55°C, and the reappearance of membrane‐localized peaks after cooling. To confirm that the disassemble phenomenon is mainly caused by the change of lipid packing of subcompartment instead of the entropy of the system rising toward a more disordered, nonassociated state by increasing temperature, we coencapsulated DMPC LUVs (*T*_m_ = 24°C) and DPPC LUVs in the POPC/POPG (70/30 mol%) GUVs in the presence of 5 mM Mg^2+^. At 15°C, DMPC LUVs and DPPC LUVs both docked to the GUVs membrane. When the temperature is increased to 37°C, DMPC LUVs detached over time while DPPC LUVs remained attached to the membrane (Figure S8A). Statistics of GUVs displaying membrane binding of DPPC and DMPC LUVs at different temperatures are shown in Figure S8B. Together, these results establish temperature as a reversible physical cue for Mg^2+^‐mediated LUVs subcompartment organization in synthetic cells.

## 3 Conclusion

This work establishes a fundamental protein‐free mechanism for dynamically organizing subcompartments within synthetic cells through Mg^2+^‐mediated membrane adhesion. We show that Mg^2+^ can reversibly bridge neutral zwitterionic and negatively charged lipid membranes, enabling the controlled formation and dissolution of subcompartment layers. In our system, LUVs act as model organelles that dock to the surface of GUVs when Mg^2+^ (5–20 mM) is present, forming a condensed membrane‐bound layer that can be disassembled by EDTA chelation and reassembled upon Mg^2+^ reintroduction. The process is reversible and selective, with adhesion strength and kinetics depending on membrane charge density, lipid phase, and temperature.

By exploiting these dependencies, we can generate a variety of hierarchical architectures within synthetic cells from uniformly coated layers to spatially segregated patches that mirror the behavior of membrane contact sites in living cells. Phase‐separated GUVs, in particular, reveal that LUVs preferentially associate with membrane domains enriched in negatively charged lipids, highlighting how ionic and compositional asymmetry can guide subcompartment positioning. Furthermore, the Mg^2+^‐mediated bridges are sensitive to the lipid phase transition. Above the gel‐to‐fluid transition temperature of DPPC subcompartment docking is lost, but reappears upon cooling. This temperature‐dependent reversibility introduces a new degree of control that integrates both chemical and physical cues into the dynamic organization of synthetic cell interiors.

Our findings provide a conceptual and experimental framework for using ion, specifically Mg^2+^‐mediated membrane interactions as a design principle for synthetic cells. The simplicity of the system, which relies solely on defined lipids, ions, and environmental modulation, makes it an accessible tool to achieve spatiotemporal regulation of subcompartments. Such control can be harnessed to mimic biological processes such as organelle docking and contact site formation, and to engineer synthetic cells that respond to environmental stimuli by reorganizing their hierarchical architecture.

By reconstructing these phenomena in a minimal context, we bridge molecular physiochemistry and synthetic biology, demonstrating that membrane‐encoded physicochemical principles alone can generate to some extent the dynamic organization that we are observing in living organisms without the need for complex protein machinery. Although the specificity of these effects is clearly limited in contrast to highly regulated protein functions, this study might offer insight into how ionic conditions, lipid composition, and thermodynamic phase transitions cooperate to modulate membrane contacts in natural systems, and alert the community of the relevance of quantitatively considering ionic conditions as key regulatory elements in all functional assays.

## 4 Experimental Section

### 4.1 Materials

Lipids including POPC, POPG, DOPC, DOPG, DPPC, DPPG, DMPC, and cholesterol were obtained from Avanti Polar Lipids (Alabaster, AL, USA) and used without further purification. Fluorescently labeled lipids: Atto655‐DOPE, Atto655‐DPPE Atto488‐DPPE, Atto488‐DMPE, and Atto488‐DOPE were purchased from ATTO‐TEC (Siegen, Germany). Lipid mixtures were prepared by codissolving the desired molar ratios of lipids in chloroform. Sucrose, calcein, Sephadex G‐50, chloroform, BSA, and mineral oil were obtained from Sigma–Aldrich (St. Louis, MO, USA). KCl was purchased from VWR (Radnor, PA, USA) and decane from TCI Deutschland GmbH (Eschborn, Germany). Magnesium chloride hexahydrate (MgCl_2_ · 6H_2_O) was purchased from Supelco (Bellefonte, PA, USA) and EDTA from PanReac AppliChem (Darmstadt, Germany). PD‐10 desalting columns were obtained from GE Healthcare (Chicago, IL, USA). All other buffer reagents were purchased from Merck (Darmstadt, Germany).

### 4.2 Construction of Synthetic Cells with Subcompartment‐Layered Architectures

Lipid films for subcompartment (LUV) and synthetic cell (GUV) formation were prepared by dissolving lipid mixtures in chloroform to generate stock solutions at 20 or 25 mg/mL. For each desired phospholipid concentration, appropriate aliquots were transferred into glass vials, and the solvent was evaporated under a gentle nitrogen stream, followed by desiccation for at least 1 hr. When required, 0.05 mol% fluorescent lipid was incorporated into the phospholipid film. DPPC LUVs and GUVs were labeled by Atto655‐DPPE or Atto488‐DPPE. DOPC LUVs were labeled by Atto655‐DOPE, DMPC LUVs were labeled by Atto488‐DMPE or Atto488‐DOPE. Unless otherwise specified, all buffers contained 150 mM KCl and 25 mM Tris (pH 7.5) and are referred to hereafter as the standard buffer.

For LUV extrusion, lipid films were rehydrated in sucrose solution (∼450 mM) prepared in the standard buffer with or without MgCl_2_, yielding a final lipid concentration of 5 mg/mL. The suspension was gently vortexed for 2 min, subjected to five freeze–thaw cycles using liquid nitrogen and a heat block, and extruded through a 200 nm polycarbonate membrane (Cytiva, Marlborough, MA, USA) above the lipid transition temperature. When encapsulating calcein, the lipid concentration was increased to 10 mg/mL, and unencapsulated dye was removed using either a Sephadex G‐50 or PD‐10 desalting column.

Homogeneous POPC/POPG GUVs were generated via the phase‐transfer method. Lipids dissolved in a decane/mineral oil mixture (3:47 v/v, 2.5 mg/mL total lipid) were sonicated in water bath for 15 min. To form inner LUV‐containing GUVs, 5 µL LUV suspension was added to 150 µL of the lipid/oil mixture and gently tapped to form an emulsion. Separately, 120 µL of lipid/oil solution was layered over 500 µL of glucose (∼450 mM) solution prepared in the standard buffer with or without MgCl_2_. The emulsion was added on top and centrifuged at 1000 rcf for 10 min. The oil layer was carefully removed, and GUVs were collected at the bottom of the tube. To form empty homogeneous GUVs, sucrose standard solution was used as the inner solution. For the timeseries experiment, inner solution of sucrose standard buffer with 5 mM MgCl_2_ and 20 mg/mL BSA was used to prevent GUVs movement.

For DPPC GUVs and phase‐separated GUVs (PS‐GUVs), vesicles were prepared by electroformation in custom‐made PTFE chambers equipped with platinum electrodes. Briefly, 6 µL of lipid mixture (2 mg/mL in chloroform) was spread on two Pt wires and air‐dried for 5 min. Chambers were filled with 350 µL of sucrose solution (∼794 mOsm/kg). An AC electric field (Aim‐TTi, Cambridgeshire, United Kingdom) of 2 rms at 10 Hz was applied for 1 h, followed by 2 Hz for 30 min at 50°C. The sample was then cooled to room temperature for > 2 h before use.

To assemble LUVs on the outer surface of GUVs, homogeneous GUVs were diluted threefold with the glucose standard buffer and mixed with LUVs at a 100:1 volume ratio, followed by 5 min incubation in the presence of 5 mM MgCl_2_. For LUV/PS‐GUV interaction, 1 µL of LUV suspension was added to 30 µL of PS‐GUV solution, followed by 169 µL of isotonic glucose standard buffer containing 5 mM MgCl_2_. The mixture was gently mixed and incubated for 5 min at room temperature before imaging.

Prepared samples with different synthetic cell architectures were loaded into ibitreat 96‐well plates (ibidi, Gräfelfing, Germany) and imaged using LSM 780, 800, or 980 inverted confocal microscopes (Carl Zeiss, Oberkochen, Germany). Image analysis was performed using ImageJ [61] and Zen Blue (Carl Zeiss, Oberkochen, Germany) software.

### 4.3 Dynamic Release and Relayering of Outer Subcompartments

For the dissociation of Atto655‐labeled and calcein‐loaded LUVs from the outer membrane of GUVs, 4 µL of 500 mM EDTA was added to 396 µL of the LUV–GUV mixture, followed by gentle vortexing and incubation for 5 min at room temperature. To re‐establish LUV layers on the GUV surface, 2 µL of 500 mM MgCl_2_ was subsequently added to 198 µL of the EDTA‐treated LUV–GUV suspension. The overall procedure is illustrated in Figure S4.

### 4.4 Thermally Induced Release and Relayering of Inner Subcompartments

Temperature control was applied to study the release and relayering of inner subcompartment layers. A custom‐made imaging chamber was assembled by stacking three imaging spacers (Sigma–Aldrich, St. Louis, MO, USA) between two clean glass coverslips (#1.5, 24 × 40 mm; Thermo Fisher Scientific, Waltham, MA, USA). The glass surface was passivated with 50 mg/mL BSA solution before sample loading. 25 µL of GUV suspension was added to the chamber, which was then mounted onto a PE120‐XY Peltier temperature control stage (Linkam Scientific Instruments, Tadworth, United Kingdom) on a Zeiss LSM 780 confocal microscope. Images were acquired using a Zeiss C‐Apochromat 20× air objective.

### 4.5 Fluorescence Intensity Profile Measurements

Fluorescence intensity profiles as a function of radius were measured using ImageJ software, and intensity profiles as a function of distance were analyzed with ZEN Blue. The data were replotted in R (version 4.4.2). For time‐lapse experiments of external binding, the 3D drift correction function in imageJ was applied to the time‐series data, which fluorescence data were replotted using Python (version 3.12.10).

### 4.6 DLS and Zeta Potential

DLS and zeta potential measurements were conducted using Zetasizer Nano ZSP (Malvern Panalytical, Malvern, United Kingdom) equipped with a 633 nm laser and a fixed backscattering angle of 173°. To determine the hydrodynamic radius, 800 µL of LUVs diluted 20‐fold was analyzed. Zeta potential measurements were carried out in a DTS1070 cell (Malvern Panalytical, Malvern, United Kingdom) using 800 µL of 20× diluted LUVs prepared in the respective buffer solution. For samples exhibiting high conductivity, data were analyzed in monomodal mode. Two experimental conditions were examined: (i) LUVs prepared in Mg^2+^‐free sucrose standard buffer and titrated with increasing Mg^2+^ concentrations (Figure S3), and (ii) LUVs directly formed in sucrose standard buffer containing 5 mM Mg^2+^ (Figure S5, S7). The data were replotted using R for visualization.

## Supporting Information

Additional supporting information can be found online in the Supporting Information section.

## Conflicts of Interest

The authors declare no conflicts of interest.

## Supporting information

Supplementary Material
