# Supplementary material for: Ion‐ and Temperature‐Programmable Reconfiguration of Subcompartments in Synthetic Cells
Source: Chembiochem. 2026 Apr 24;27(8):e202500928. doi: 10.1002/cbic.202500928 (PMC13108542; doi:10.1002/cbic.202500928)
Supplement: Supplementary file 1 — Supplementary Material [file CBIC-27-e202500928-s001.zip › cbic70257-sup-0001-SuppData-S1/3_20260227_MgSub_supplementary_proof.pdf]

Supporting Information for

Ion- and Temperature-Programmable Reconfiguration of Subcompartments in Synthetic Cells

Figure S1–S2. Calcein encapsulation and control experiments.

Figure S3. Dynamic light scattering (DLS) and zeta potential characterization of DPPC and POPC/POPG vesicles.

Figure S4. Schematic illustration of EDTA-mediated Mg^{2+} chelation process.

Figure S5. Zeta potential characterization of POPC/POPG vesicles as a function of POPG mol% at 5 mM Mg^{2+}

Figure S6. Quantitative analysis of GUVs displaying DPPC subcompartment layer at different POPG concentration

Figure S7. DLS and zeta potential analysis of DOPC and DPPC vesicles.

Figure S8. Confocal imaging and quantitative analysis of co-encapsulated DMPC and DPPC LUVs.

Video S1. Kinetic of DPPC LUVs bind to POPC/POPG (70/30 mol%) GUVs membrane in the present of 5 mM Mg^{2+}.

Video S2. Reversible temperature-controlled docking of DPPC subcompartments to POPC/POPG (70/30) GUVs in 5 mM Mg^{2+}. DPPC LUVs attach below the phase transition temperature, detach upon heating, and reassemble when cooled below T_m.

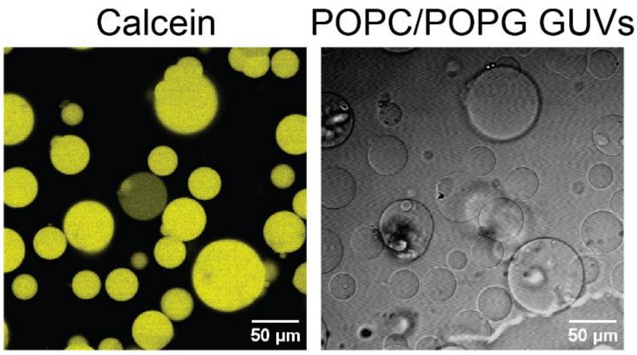

Figure S1. Calcein encapsulated in the POPC/POPG (70/30 mol%) GUVs in the present of 5 mM Mg^{2+}.

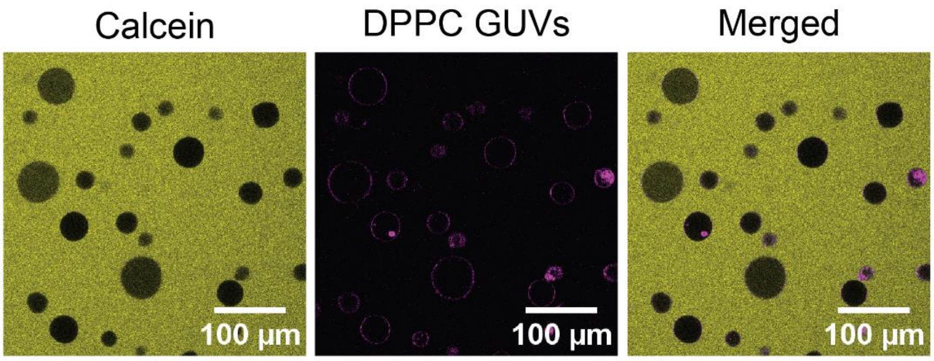

Figure S2. Calcein added from the outside of Atto655-DPPE labeled DPPC GUVs in the present of 5 mM Mg^{2+}.

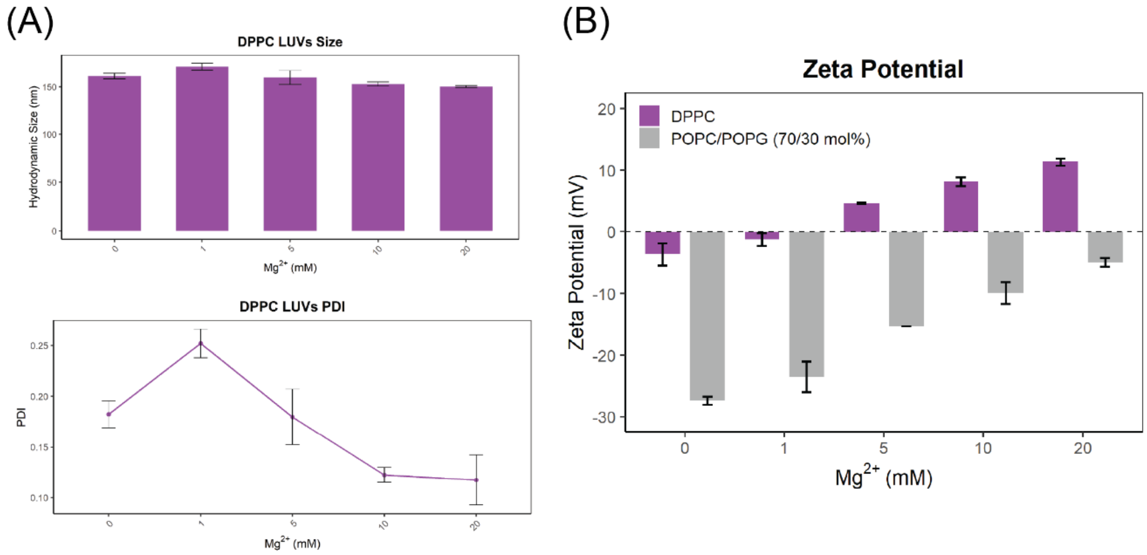

Figure S3. (A) Hydrodynamic diameter (top) and polydispersity index (bottom) of DPPC LUVs measured at varying Mg^{2+} concentrations (n=2, mean ± SD). (B) Zeta potential of DPPC LUVs and POPC/POPG (70/30 mol%) LUVs measured at various Mg^{2+} concentrations (n=2, mean ± SD). LUVs were prepared in Mg^{2+}-free buffer and subsequently titrated with increasing Mg^{2+} concentrations to measure changes in hydrodynamic diameter and zeta potential.

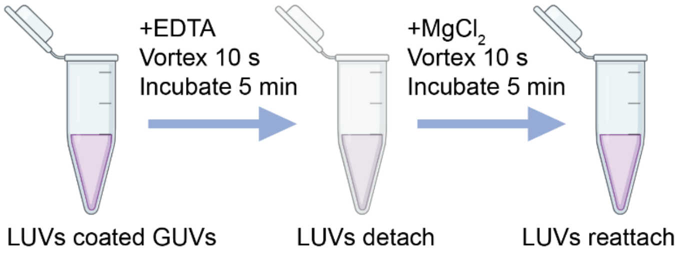

Figure S4: Scheme depicting the release and re-layering of sub-compartments on the outer synthetic cell membrane. Created in BioRender. Xu, Z. (2026) https://BioRender.com/vfjks6x

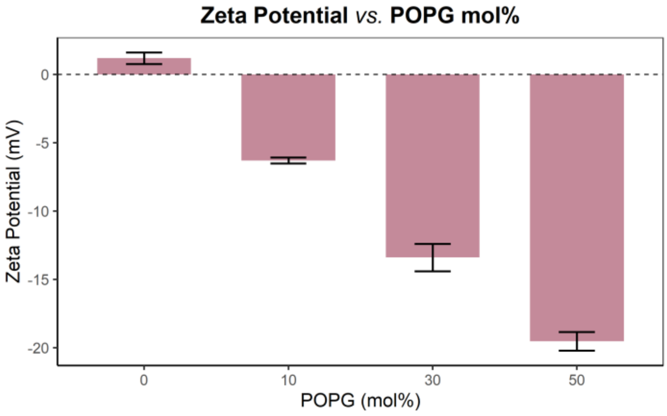

Figure S5: Zeta potential of POPC/POPG LUVs as a function of POPG mol% at 5 mM Mg^{2+} (n = 3, mean ± SD).

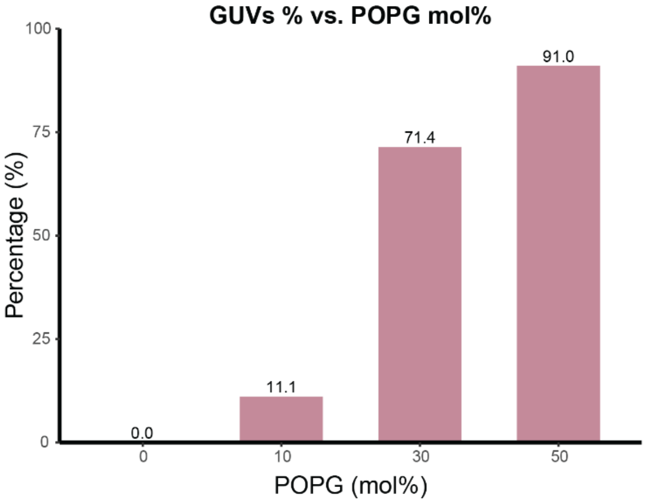

Figure S6. Quantification of GUVs displaying DPPC subcompartment layer at POPG concentration of 0 mol% (n = 134), 10 mol% (n = 199), 30 mol% (n=318) and 50 mol% (n = 301).

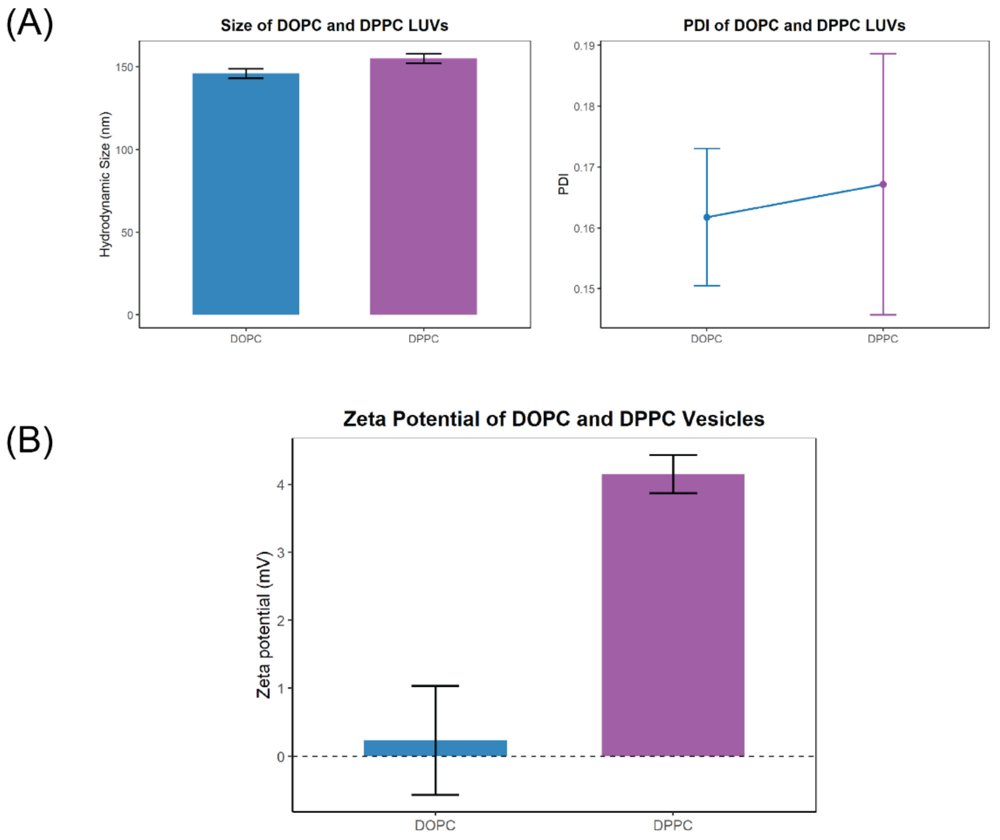

Figure S7: (A) Hydrodynamic diameter (left) and polydispersity index (right) of DOPC and DPPC LUVs measured at 5 mM Mg^{2+} concentrations (n=3 for DOPC, n=2 for DPPC, mean ± SD). (B) Zeta potential of DOPC and DPPC LUVs measured at 5 mM Mg^{2+} concentrations (n=3 for DOPC, n=2 for DPPC, mean ± SD). LUVs were directly prepared in sucrose standard buffer containing 5 mM Mg^{2+}.

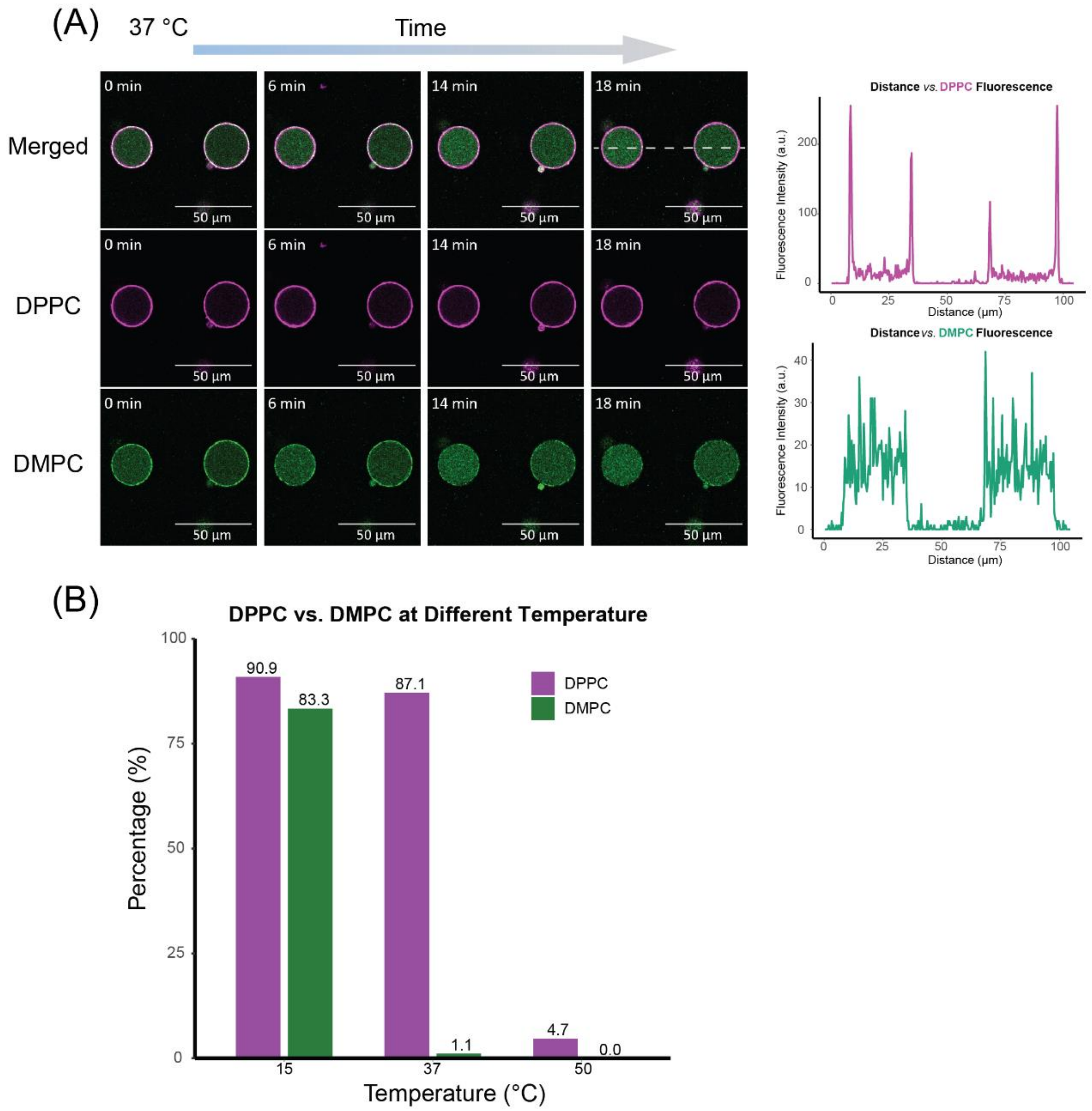

Figure S8. (A) Time-lapse confocal images showing co-encapsulated DPPC and DMPC LUVs (1:1 v/v) labeled with Atto655-DPPE and Atto488-DOPE, respectively, in the presence of 5 mM Mg^{2+} at 37 °C (left). The corresponding fluorescence intensity profiles at 18 min are plotted as a function of distance (right). (B) Quantification of GUVs displaying membrane binding of DPPC and DMPC LUVs at 15 °C (n = 66), 37 °C (n = 101), and 50 °C (n = 85).
